# Socioeconomic, demographic, and cultural determinants of delivery by caesarian section in Ethiopia: Evidence from Ethiopia Mini Demographic and Health Survey 2019

**DOI:** 10.1371/journal.pone.0288022

**Published:** 2023-07-06

**Authors:** Mohammad Omar Faruk, Md. Eyasin Arafat, Sabbir Hussain Shanta

**Affiliations:** Department of Statistics, Noakhali Science and Technology University, Noakhali, Bangladesh; Flinders University, AUSTRALIA

## Abstract

Delivery by cesarean section is a surgical procedure of delivery to a newborn baby, and the process is applied when vaginal delivery is unsafe. This study aims to identify the socioeconomic, demographic, and cultural factors that significantly impact the delivery by caesarean section. The 2019 Ethiopia Mini Demographic and Health Survey (2019 EMDHS) data were used to conduct this research, and this study considered 2872 ever-married women all over the country who delivered in the clinical setting. Firstly, a frequency distribution table has been constructed to understand the characteristics of the selected explanatory and study variables. Then Chi-square test identifies the association between various socioeconomic and demographic factors and delivery by the caesarian section. Finally, the Binary Logistic Regression was used to determine the factors that substantially impact the caesarian section among women in Ethiopia. The Chi-square test of association showed that mother’s age, type of residence, highest education level, religion, socioeconomic status, total children ever born, use of contraception, age of mothers at first birth, and preceding birth interval were significantly associated with the caesarian section. The multivariate binary logistic regression analysis revealed that the mother’s current age (Age Group: 31–40; Odds Ratio: 2.487, p<0.05) and religion (Muslim; Odds Ratio: 0.599, p<0.05) substantially influenced the study feature. Moreover, the highest educational level (Secondary and higher; Odds Ratio: 1.581, p<0.05), and the preceding birth interval (>40 months; Odds Ratio: 0.682, p<0.05) were also found to have considerable impacts on the caesarian section. Furthermore, the total number of children ever born (>5; Odds Ratio: 0.498, p<0.05) significantly impacts the delivery by caesarean section in Ethiopia. This study’s results would be useful to policymakers to take necessary steps to reduce unnecessary delivery by caesarian section and ensure a safer newborn delivery process.

## Introduction

Delivery by Cesarean section (DCS) is a life-saving surgical procedure for both the mother and baby and is delivered via an incision in the mother’s abdomen. In the past two decades, global cesarean section rates have steadily risen [1]. Therefore, short-term and long-term maternal and neonatal complications are increasing. This study aims to identify the socioeconomic, demographic, and anthropometric factors affecting caesarian section delivery in Ethiopia. Historically, the Cesarean section (CS) has played a significant role in obstetrics and human culture when vaginal delivery is either impossible or poses excessive risks to the mother or the newborn [2]. WHO has repeatedly stated that DCS rates should not exceed 15% based on population [3]. DCS is the most commonly performed significant operation worldwide; more than 1 million DCS are performed annually in the United States of America (USA) alone [4]. There were also high DCS rates in developing countries like Ethiopia, ranging from 21.1% to 34.3% [5–7]. DCS rates have increased substantially due to unnecessary operations attributable to non-evidence-based indications, professional convenience, maternal requests, and monetary considerations worldwide [8]. An analysis of cesarean delivery rates in 137 countries found that cesarean delivery rates varied significantly worldwide. The cesarean delivery rate was less than 10% in 54 countries, between 10 and 15% in 14 countries, and higher than 15% in 69 countries [9]. The WHO publications indicate that between 1990 and 2014, the global average DCS rate rose from 12.4% to 18.6%, varying by region from 6.2% to 27.2% and increasing by 4.4% annually [10]. Research has indicated that DCS is being provided at higher rates than recommended, both in high- and low-income countries. The lowest rates were found in Africa (7.3%), followed by Asia (19.2%), Europe (25%), Oceania (31.1%), and North America (32.3%) [1]. While all the other regions showed an increase in DCS, there was a slight but real increase in the DCS rates in sub-Saharan Africa (SSA) [1]. Women’s attitudes toward childbirth and delivery have changed considerably in recent years. Instead of having a positive attitude toward vaginal delivery, many women request caesarian deliveries for non-medical reasons, which is an unhealthy trend [11]. In Ethiopia, maternal and neonatal mortality rates remain high, with 37 neonatal deaths per 1000 live births [12]. While the institutional delivery rate is increasing nationwide, efforts to reduce maternal and neonatal mortality have not led to significant changes [12]. Several socioeconomic, demographic, and cultural factors influence caesarean section [13–15]. Literature shows that maternal age and the number of children affect DCS [16, 17]. The mothers ’ socioeconomic status and place of residence also play a substantial role in performing DCS [17, 18]. Maternal education significantly impacts the caesarean section, and mothers with higher education levels are more prone to choose CS than mothers with low education levels [14, 18, 19]. Even though limited studies are performed on caesarean section in Ethiopia, there is a lack of evidence on the scope and predictors of cesarean section in the study area, which is very important to identify and avoid causes for the dramatic increase in cesarean section rates. Therefore, this study aimed to assess the factors influencing cesarean section delivery among women in Ethiopia. The results of this study help us understand the influential factors and decision-making processes that make it easier to modify the overall DCS rates.

## Materials and methods

### Study area and data collection

This study aimed to identify the determinants of DCS in Ethiopia. It is landlocked and one of the countries in the Horn of Africa [20, 21]. The country lies in north-south and east-west dimensions, relatively compact and entirely within tropical latitudes. The capital’s name is Addis Ababa, and this city is located in the centre of the country. It is Africa’s largest and 2nd most populated country [22]. The data for this investigation were collected from the 2019 Ethiopia Mini Demographic and Health Survey (2019 EMDHS) children’s records [23]. The Ethiopian Public Health Institute (EPHI) implemented the 2019 Ethiopia Mini Demographic and Health Survey (2019 EMDHS) along with the Federal Ministry of Health (FMoH) and Central Statistical Agency (CSA), and the overall guidance was provided by the Technical Working Group (TWG). The Data was collected from March to June 2019. The World Bank, the United States Agency for International Development (USAID), and the United Nations Children’s Fund (UNICEF) funded the 2019 EMDHS. Technical assistance for the DHS Program was provided by ICF, a USAID-funded project providing technical assistance and support in implementing demographic and health surveys across countries worldwide. All the figures and tables used in this study have been constructed by the authors of this research based on 2019 EMDHS data [23].

### Dependent variable

The dependent variable considered in this study was delivery by the caesarian section (No, Yes), a binary variable. Mothers who gave at least one birth with CS were categorized as “Yes”, and those with no CS were classified as “No”.

### Explanatory variables

A total of 13 independent variables have been considered in this study. The explanatory variables were divided into three different groups: socioeconomic, demographic, and cultural features. The socioeconomic variables include socioeconomic status (Poor, Middle, Rich), type of residence (Urban, Rural), and educational level (No education, Primary, Secondary and above). The demographic group consists of the age of the mothers (15–20, 21–30, 31–40, 41–49), total children ever born (1–2, 3–5, >5), sons have died (No, Yes), use of contraception (No, Yes), age of mothers at 1^st^ birth (10–15, 16–20, 21–25, 26–30, 31–36, 36–44), currently breastfeeding (No, Yes), sex of the child (Male, Female), the child is alive (No, Yes), and preceding birth interval (10–20, 21–30, 31–40, >40). Finally, the cultural feature was the religion (Orthodox, Protestant, Muslim, Other). Initially, the mother’s current age, the total number of children ever born, the age of mothers at 1st birth, and the preceding birth interval were discrete numeric variables; later, they were converted to categorical features as mentioned above as the study’s dependent variable is a binary categorical variable and make a better presentation of the interpretations.

### Study population

The 2019 EMDHS children record data considered a total of 5753 women, of which 2881 gave birth at home, and 2872 delivered their baby at a health facility setting. The study population of this investigation was the women who delivered in the clinical setting (n = 2872) in Ethiopia. These women were asked about DCS, and 12.2% (n = 349) of women were found to give birth by cesarean section (Table 1). This study excluded all women who gave birth at home setting.

**Table 1:** Percentage distribution of the respondents by background characteristic of caesarian section in Ethiopia.

Variables	Categories	Frequency	Percent
Mother’s current age	15–20	331	11.5
	21–30	1722	60
	31–40	720	25.1
	41–49	99	3.4
Type of place of residence	Urban	1104	38.4
	Rural	1768	61.6
Highest educational level	No education	1066	37.1
	Primary	1116	38.9
	Secondary and higher	690	24
Religion	Orthodox	1013	35.3
	Protestant	510	17.8
	Muslim	1303	45.4
	Other	46	1.6
Socio Economic Status	Poor	878	30.6
	Middle	882	30.7
	Rich	1112	38.7
Total children ever born	1–2	1318	45.9
	3–5	1017	35.4
	>5	537	18.7
Sons who have died	No	2471	86
	Yes	401	14
Use of contraceptive method	Not using	1608	56
	Using	1264	44
Currently breastfeeding	No	1123	39.1
	Yes	1749	60.9
Sex of child	Male	1468	51.1
	Female	1404	48.9
Child is alive	No	142	4.9
	Yes	2730	95.1
Preceding birth interval (months)	10–20	1107	38.5
	21–30	431	15
	31–40	410	14.3
	>40	924	32.2
Age of mothers at first birth	10–15	555	19.3
	16–20	1334	46.4
	21–25	770	26.8
	26–30	145	5
	31–36	59	2.1
	36–44	9	0.3
Delivery by caesarean section	No	2523	87.8
	Yes	349	12.2

### Statistical analysis

The data for this study have been extracted from the 2019 Ethiopia Mini Demographic and Health Survey (2019 EMDHS). The total number of respondents to the survey was 5753. This study considered 2872 respondents who gave birth at the health facility setting to assess the impact of demographic, socioeconomic, and cultural factors on DCS. It is observed that 2523 (87.8%) respondents delivered their child in a normal way, and 349 (12.2%) respondents delivered their child in a caesarian way. Initially, 13 explanatory variables were considered in this study that potentially influenced the dependent variable "delivery by caesarian section". Firstly, the frequency distribution of all the dependent and independent variables has been prepared to gain knowledge about the background characteristics of the respondents. Since the outcome variables considered in this study were categorical, the non-parametric statistical approach was applied throughout the study. The non-parametric Chi-square (χ2) test was applied to identify the significant association between the delivery by caesarian section and a variety of independent variables. The explanatory variables found significant in bivariate analysis were also considered in multivariate analysis. As the study variable considered in this research is binary, the binary logistic regression model was applied as a multivariate analysis to determine the factors that significantly impact the delivery by the caesarian section in Ethiopia. A significant level of 5% (0.05) is considered for both bivariate and multivariate analysis. The Hosmer-Lemeshow’s (HL) goodness of fit test has been applied to understand the model’s acceptability used in this analysis. The HL test (Chi-square = 5.185, p-value = 0.788) proves that the model applied in this study is appropriate and better fits the research data. The study used statistical software SPSS version 25 to conduct all the statistical analyses.

### Ethical consideration

The Demographic and Health Survey (DHS) program granted this research to collect Ethiopian Mini Demographic and Health Survey 2019 (EMDHS 2019) data after carefully reviewing the short description provided by the authors, and high confidentiality was maintained when dealing with the data. The ethical approval for EMDHS 2019 was obtained from the Ministry of Science and Technology, IFC International’s Institutional Review Board, the Ethiopian Health and Nutrition Institute Review Board, and the CDC. Informed consent was obtained from the respondents when collecting the data, ensuring the information would be kept confidential [24].

## Results

**Fig 1:**
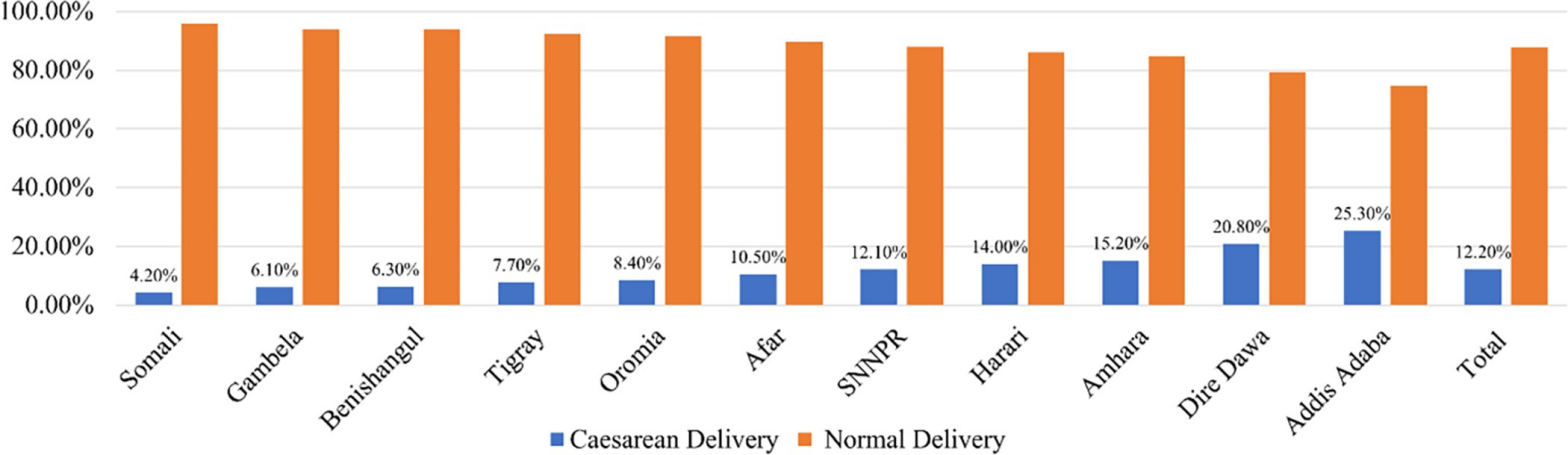
Percentage of DCS in different regions of Ethiopia.

Fig 1 illustrates the percentage of CS delivery in different regions of Ethiopia. The figure showed that the most prevalent area in Ethiopia is Addis Ababa (25.30%), followed by Dire Dawa (20.80%). The minimum percentage of DCS was observed in Somali (4.20%), following Gambela (6.10%). The overall percentage of CS in clinical settings in Ethiopia is 12.20%.

**Fig 2:**
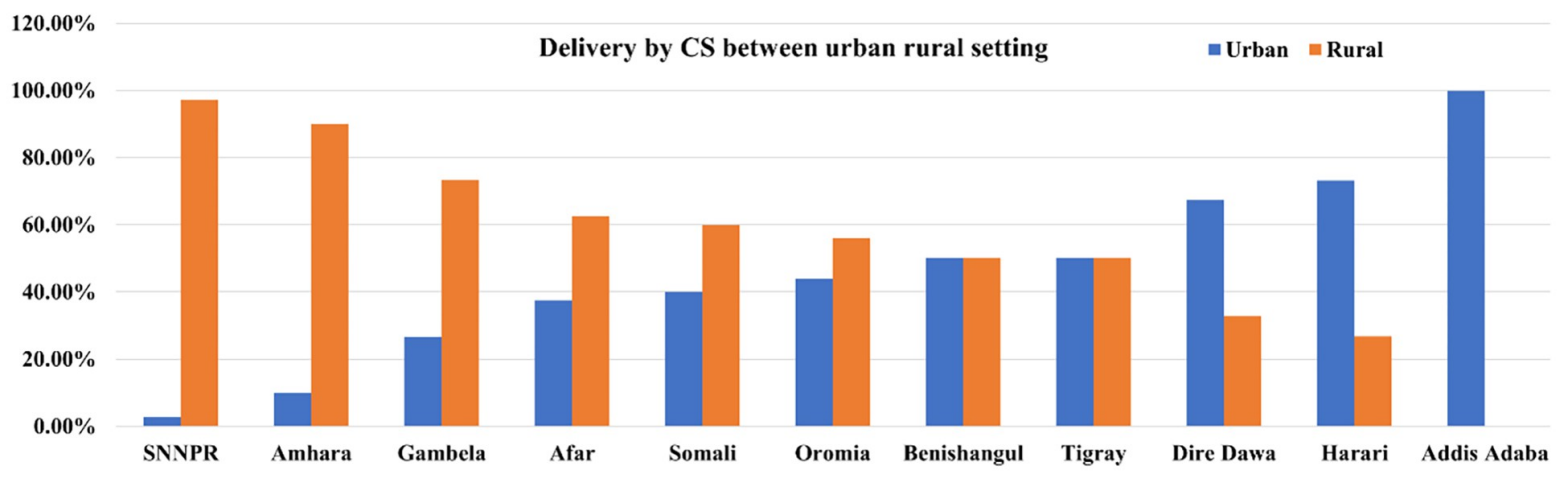
Percentage of CS in urban-rural settings among different regions of Ethiopia.

From Fig 2 it is observed that Addis Ababa (100%), Harari (73.20%), and Dire Dawa (67.30%) had a greater percentage of DCS in urban areas. On the other hand, Southern Nations, Nationalities, and People’s Region (SNNPR) (97.20%), Amhara (90%), and Gambela (73.30%) had the maximum caesarean delivery in rural areas. The figure also revealed that Benishangul and Tigray had almost fifty-fifty of the CS in urban and rural areas.

**Fig 3:**
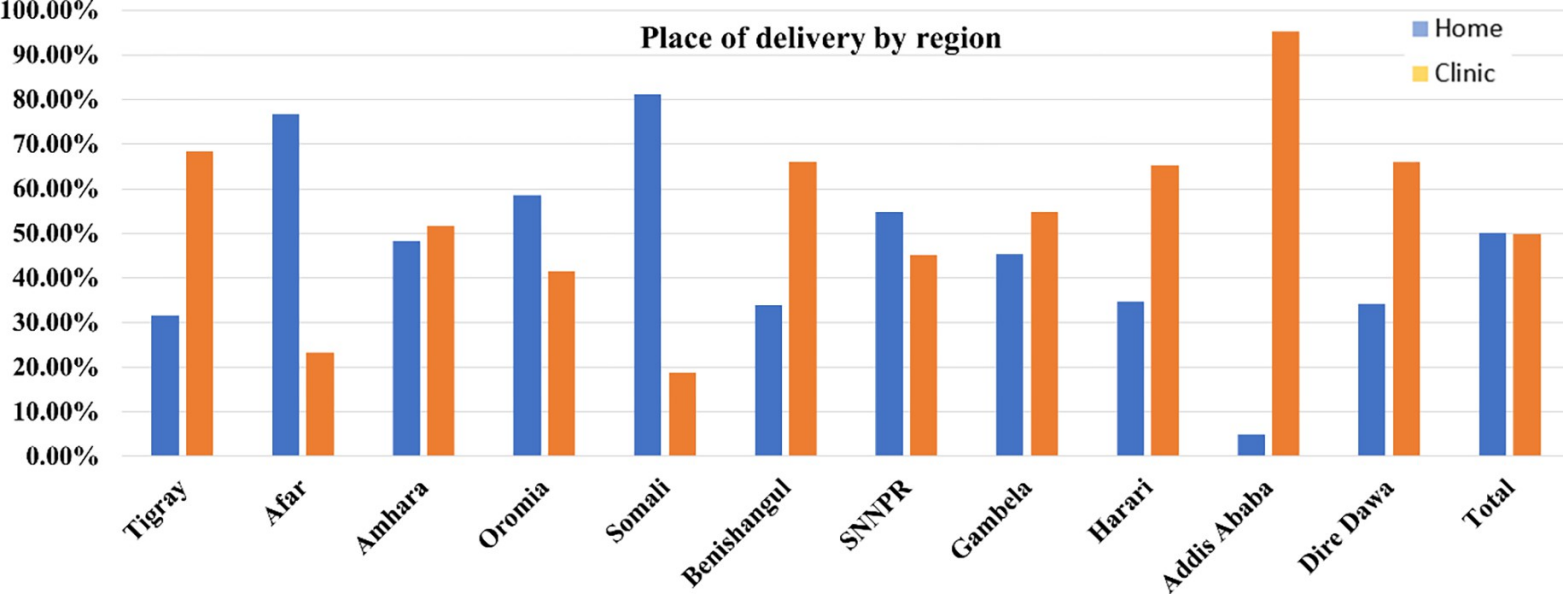
Percentage of deliveries in different regions of Ethiopia at home and clinical settings.

In addition, Fig 3 illustrates the percentage distribution of deliveries at home and clinics in various parts of Ethiopia. The percentage of clinical (49.9%) and home delivery (50.1%) is almost the same. Clinical delivery was found to be high in Addis Ababa (95.20%), followed by Tigray (68.30%), Benishangul (66.00%), and Dire Dawa (65.90%). In contrast, the maximum of home deliveries are found in Somali (81.20%), following Afar (76.70%) and Oromia (58.60%).

A frequency distribution table has been constructed in univariate analysis to understand the characteristics of Ethiopian women’s demographic, socioeconomic, and cultural conditions and is presented in Table 1. From Table 1, it is observed that most respondents belong to the age group 21–30 years (n = 1722, 60%), and most of the respondents (1768, 61.6%) live in rural areas. Education is essential for every part of human life, but a high percentage of the mothers (n = 1116, 38.9%) had primary education, followed by illiterate (n = 1066, 37.1%) in Ethiopia. Only a few mothers had secondary and higher education (n = 690, 24%). In the case of religion, Table 1 shows that most of the mothers are Muslim (n = 1303, 45.4%), and about (n = 1112, 38.7%) of the respondents who went to the clinic were rich.

The table illustrates that (n = 1608) 56% of the respondents do not use contraception. A significant number of women (n = 2730, 95.1%) reported that their children were alive. Preceding birth intervals play an important role in the caesarian section among women, and a maximum (n = 1107, 38.5%) of the mothers take 10–20 months from one birth to another. The variable "Age of mothers at first birth" measures the age from when girls begin childbearing. Of the maximum number of respondents (n = 1334, 46.4%) give birth at 16–20 years. The variable socioeconomic condition potentially influences the caesarian section delivery among women in Ethiopia. In addition to the univariate analysis, the bivariate analysis was performed to test the association between various explanatory variables and delivery by caesarian section among the women in Ethiopia. The Chi-square test was applied to assess these associations and presented in Table 2. The table revealed that the age of the mothers was significantly associated (Chi-square = 23.74, p<0.05) with the delivery by the caesarian section in Ethiopia.

**Table 2:** Cross-tabulation and associated summary statistics for delivery by caesarian section in Ethiopia.

Variables	Categories	Delivery by CS		Chi-Square	
		No (%)	Yes (%)		
Mother’s current age	15–20	302 (91.20)	29 (8.80)		
	21–30	1539(89.40)	183(10.60)		
	31–40	596(82.80)	124(17.20)	24.74	0.000
	41–49	86(86.90)	13(13.10)		
Type of place of residence	Urban	916(83.00)	188(17.00)		
	Rural	1607(90.90)	161(9.10)	39.96	0.000
Highest educational level	No education	966(90.60)	100(9.40)		
	Primary	1009(90.40)	107(9.60)	60.45	0.000
	Secondary and higher	548(79.40)	142(20.60)		
Religion	Orthodox	833(82.20)	180(17.80)		
	Protestant	458(89.80)	52(10.20)		
	Muslim	1188(91.20)	115(8.80)	47.89	0.000
	Other	44(95.70)	2(4.30)		
Socio-economic Status	Poor	805(91.7)	73(8.3)		
	Middle	805(91.3)	77(8,7)	56.15	0.000
	Rich	913(82,1)	199(17.9)		
Total children ever born	1–2	1122(85.10)	196(14.90)		
	3–5	909(89.40)	108(10.60)	18.52	0.000
	>5	492(91.60)	45(8.40)		
Sons who have died	No	2165(87.60)	306(12.40)		
	Yes	358(89.30)	43(10.70)	0.89	0.196
Use of contraception	Not using	1444(89.80)	164(10.20)		
	Using	1079(85.40)	185(14.60)	13.95	0.000
Currently breastfeeding	No	969(86.30)	154(13.70)		
	Yes	1554(88.90)	195(11.10)	4.21	0.024
Sex of child	Male	1290(87.90)	178(12.10)		
	Female	1233(87.80)	171(12.20)	0.002	0.505
Child is alive	No	116(81.70)	26(18.30)		
	Yes	2407(88.20)	323(11.80)	5.307	0.019
Preceding birth interval (months)	10–20	947(85.50)	160(14.50)		
	21–30	382(88.60)	49(11.40)		
	31–40	368(89.80)	42(10.20)	9.207	0.027
	>40	826(89.40)	98(10.60)		
Age of mothers at first birth	10–15	495(89.20)	60(10.80)		
	16–20	1223(91.70)	111(8.30)		
	21–25	654(84.90)	116(15.10)		
	26–30	114(78.60)	31(21.40)	109.1	0.000
	31–36	32(54.20)	27(45.80)		
	36–44	5(55.60)	4(44.40)		

From Table 2, it is observed that the type of place of residence (Chi-square = 39.96, p<0.05), educational level (Chi-square = 60.45, p<0.05), religion(Chi-square = 47.89, p<0.05), and socioeconomic status (Chi-square = 56.15, p<0.05) were significantly associated with the DCS. The table also showed that the total number of children ever born (Chi-square = 18.52, p<0.05), use of contraception (Chi-square = 13.95, p<0.05), currently breastfeeding (Chi-square = 4.21, p<0.05), preceding birth interval (Chi-square = 9.207, p<0.05), child is alive (Chi-square = 5.307, p<0.05) and age of mothers at first birth (Chi-square = 109.1, p<0.05) substantially associated with the DCS.

### Factors influencing delivery by caesarian section

The binary logistic regression is used as a multivariate analysis to determine the factors that significantly impact the DCS in Ethiopia and is presented in Table 3. From Table 3, it is observed that the mother’s current age significantly influences the DCS. The age groups 31–40 and 21–30 are 2.487 (Odds: 2.487, p<0.05) and 1.221 (Odds: 1.221, p<0.05) times, respectively, more likely to have DCS compared to the age group 15–20 years.

**Table 3:** Binary logistic regression determining the influential factors of delivery by caesarian section in Ethiopia.

Variables	B	S.E.	Exp(B)	95 C.I.for EXP(B)		Sig.
				Lower	Upper	
**Mother’s current age**						
15–20			1			0.002
21–30	0.199	0.247	1.221	0.752	1.982	0.420
31–40	0.911	0.322	2.487	1.324	4.672	0.005
41–49	0.902	0.476	2.465	0.97	6.264	0.058
**Type of place of residence**						
Urban			1			
Rural	-0.148	0.188	0.863	0.597	1.246	0.431
**Highest educational level**						
No education			1			0.002
Primary	-0.078	0.165	0.925	0.67	1.279	0.638
Secondary and higher	0.458	0.183	1.581	1.104	2.265	0.013
**Religion**						
Orthodox			1			0.001
Protestant	-0.366	0.181	0.694	0.486	0.989	0.043
Muslim	-0.512	0.141	0.599	0.454	0.791	0.000
Other	-1.235	0.744	0.291	0.068	1.249	0.097
**Socio Economic Status**						
Poor			1			0.173
Middle	-0.184	0.18	0.832	0.585	1.184	0.307
Rich	0.202	0.228	1.223	0.782	1.914	0.378
**Total children ever born**						
1–2			1			0.036
3–5	-0.324	0.18	0.723	0.508	1.029	0.072
>5	-0.698	0.273	0.498	0.291	0.85	0.011
**Use of contraceptive method**						
Not using			1			
Using	0.246	0.126	1.279	0.999	1.637	0.051
**Currently breastfeeding**						
No			1			
Yes	-0.07	0.126	0.933	0.729	1.194	0.581
**Child is alive**						
No			1			
Yes	-0.718	0.241	0.488	0.304	0.782	0.003
**Preceding birth interval (months)**						
10–20			1			0.108
21–30	0.04	0.199	1.04	0.704	1.537	0.842
31–40	-0.105	0.212	0.9	0.594	1.363	0.619
>40	-0.382	0.18	0.682	0.48	0.971	0.034
**Age of mothers at first birth**						
10–15			1			0.001
16–20	-0.605	0.184	0.546	0.38	0.784	0.001
21–25	-0.33	0.213	0.719	0.473	1.092	0.122
26–30	-0.336	0.308	0.714	0.391	1.307	0.275
31–36	0.454	0.396	1.574	0.725	3.419	0.251
36–44	0.764	0.769	2.148	0.476	9.687	0.32
Constant	-0.886	0.406	0.412			0.029
**Hosmer and Lemeshow goodness of fit test**			**Chi-square = 5.185**		**p-value = 0.788**	

Table 3 revealed that the mothers’ educational level significantly impacts DCS among Ethiopian women. The result showed that the secondary and higher educated respondents have 1.581 times (Odds: 1.581, p<0.05) more odds of DCS than their illiterate counterparts. In addition, religion is found to impact the study variable (p<0.05) significantly, and the people who belong to Orthodox are more prone to give birth by caesarian section. The total number of children born substantially impacts Ethiopia’s DCS (Odds: 0.498, p<0,05). The results showed that couples with more than 5 children were 51% less likely to have a child by caesarian section (Odds: 0.49, p<0.05) compared to their counterparts with 1–2 children. The most important factor considered in this study is the age of the mothers at first birth, and this feature is found to have a significant impact on the DCS. The study results showed that the respondents aged 36–44 years have 2.148 times (Odds: 2.148, p<0.05) more risk of having a caesarean child than mothers aged 15–20. Moreover, the variable child is alive had a substantial role in DCS (Odds: 0.488, p<0.05). The preceding birth interval significantly affected DCS (p<0.05). The odds ratio of 0.682 indicates that the couples with preceding birth intervals between babies over 40 months were 31.8% less likely to have a baby with CS than their counterparts of preceding intervals 10–20 months. The Hosmer-Lemeshow test has measured the goodness of fit of the proposed model. The Chi-square statistic and p-value in the Hosmer-Lemeshow goodness of fit test (Chi-square: 5.185, p = 0.788) indicate that the binary logistic regression fits the data well and provides more appropriate outcomes.

## Discussion

This study investigated the determinants of DCS among Ethiopian women. It is observed from the results that the mothers’ age significantly impacts the DCS, and the age group 31–40 and 41–49 had a higher likelihood of having DCS. This is because a range of diseases could develop within the body of older women, which cause various health complications and lead those women to choose caesarean delivery. Similar to the results of this investigation, Joseph A. Adashek et al. (1993) found that maternal age was significantly associated with DCS [25]. A study conducted in Ontario, Canada, by G. Janoudi, and colleagues revealed that the prevalence of the risk factors of the cesarean section increased with advancing maternal age [26]. Older women (more than 34 years) tend to choose DCS compared to women of younger age [27]. Moreover, an investigation in Denmark between 1998 and 2015 observed that women aged 35–39 years and over 40 years have twice and even tripled the risk of CS, respectively, compared to mothers younger than 30 years [28].

The present study found an interesting result that secondary and higher educated mothers are more likely to have DCS than those who are illiterate. Highly educated women are aware of their health and concerned about the safety of health risk perception. Moreover, access to biased information and positive attitude towards caesarean birth are the reasons for preferring the DCS [11]. In line with the results of the current study, a meta-analysis focused on Sub-Saharan Africa revealed that maternal education was found to affect the delivery by cesarean section [20] significantly. In addition, women with a higher level of education are more susceptible to choosing DCS [29, 30]. A study measuring the trend of cesarean section in Turkey observed that the rate of CS increase with the increase in mothers’ education levels [31]. Moreover, a study of the global epidemiology of use of and disparities in CS revealed substantial differences in CS use within countries, especially among more educated women [32]. In contrast, several studies also found contradictory results that women with lower education are more likely to select a cesarian section [33, 34].

This study revealed that religion substantially impacted the caesarean section in Ethiopia, and Muslims had the lowest likelihood of having CS compared to Orthodox and Protestants. A survey in a public university hospital in Dar es Salaam, Tanzania, found that religious belief significantly influences women’s caesarean section attitude [35]. Moreover, social and cultural variables like education, religion, and wealth index were found to be substantial with DCS in several studies [34, 36]. A qualitative study in Nigeria showed that religion plays a vital role in the caesarian section [37]. Furthermore, the religious provider influences the women regarding antenatal care (ANC) and the delivery landscape by promising ‘faith’ and ‘divine protection’ based outcomes other than child birthing skills [38, 39], which leads women to a perilous situation. The total number of children born per woman is significantly associated with the cesarian section in Ethiopia. This study found that women with few children are more prone to have a delivery by caesarian section. A study in Bangladesh found that the total number of children and age at first birth are the most important factors for the cesarian section [40]. An investigation performed in sub-Saharan African countries revealed a significant reduction in CS with a per unit increase in the number of children [41]. A study found contradictory results to ours; women with numbers of babies less than 2 are associated with low caesarean section [42].

The mother’s age at first birth was also found to be significant with CS in this country, and the women who give their first birth at a higher age were more susceptible to DCS than younger women. The study conducted in the UK observed that young women’s age at first birth seemed to be protective of the later caesarian section [43]. A report from 1996 to 2021 in the United States of America (USA) leads to the same conclusion as the present study that a higher rate of CS was observed among the mother whose age at first birth was high (40–54 years) and lower CS rate (19.4%) was found among younger mothers [44]. A study based on Bangladesh Demographic and Health Survey 2014 (BDHS 2014) revealed that mothers whose age at first birth is greater than 20 years are more prone to have DCS following being overweight or obese [40]. The livelihood of the child also influences DCS. This research showed that mothers whose child is alive have a lower risk of having a baby with DCS compared to mothers who experienced their child’s death. Mothers who had a previous bad experience with their children’s death were more careful about their later delivery and were unwilling to take risks rather than safe childbirth, and chose to use DCS. A study in Ethiopia from nationally representative EDHS from 2000 to 2016 revealed a strong association between neonatal death/alive and CS [45].

This study found that the preceding birth interval significantly influences DCS in Ethiopia, and with the increase in the birth interval, the risk of having DCS tends to decrease. The mothers with preceding birth intervals of 21–30 months are more susceptible to DCS. An investigation identifying the prevalence and determinants of CS in Alexandria, Egypt, elucidated that preceding birth intervals substantially impact the DCS among Egyptian women [46]. A systematic review and meta-analysis on CS and pregnancy interval observed the association of birth interval with DCS [47]. Women with more preceding birth intervals had more likelihood of undergoing CS [48]. However, in addition to the features considered in this study, there may be other influential factors like maternal physical conditions, public-private health facility setting, and different environmental factors that could potentially affect the DCS, which is not included in this study and is the limitation of the present investigation. This investigation suggests further research determining the mentioned feature’s importance in DCS. Moreover, the strength of this study is that the data used in this study are nationally representative, and the investigation accurately reports the statistical significance of the effect of various socio-economic, demographic, and cultural determinants on DCS.

## Conclusion

In both developed and developing countries, caesarian deliveries have increased for decades. However, Ethiopia still lacks this service, which could mean missing out on potentially life-saving opportunities. Women’s current age, educational status, religion, total children ever born, age of mothers at 1st birth, and preceding birth interval significantly impacted the delivery by caesarian section. Policy decisions will be needed for further national-level interventions depending on the results of this study. These factors should also be considered in health promotion programs to reduce unnecessary CS; Governments need to take the necessary steps to reduce caesarean rates and increase natural and healthy newborn delivery.
